# Automated Bird Counting with Deep Learning for Regional Bird Distribution Mapping

**DOI:** 10.3390/ani10071207

**Published:** 2020-07-16

**Authors:** Hüseyin Gökhan Akçay, Bekir Kabasakal, Duygugül Aksu, Nusret Demir, Melih Öz, Ali Erdoğan

**Affiliations:** 1 Department of Computer Engineering, Akdeniz University, Antalya 07058, Turkey; hgakcay@akdeniz.edu.tr (H.G.A.); melihoz@akdeniz.edu.tr (M.O.); 2 Department of Biology, Akdeniz University, Antalya 07058, Turkey; kabasakalbekir@gmail.com (B.K.); aerdogan@akdeniz.edu.tr (A.E.); 3 Anesthesia Programme, Department of Medical Services and Techniques, Vocational School of Health Services, Antalya Bilim University, Antalya 07190, Turkey; 4 Department of Space Science and Technologies, Akdeniz University, Antalya 07058, Turkey; duygugulaksu@gmail.com

**Keywords:** computer vision, machine learning, deep learning, bird detection, bird counting, bird monitoring, bird population mapping, bird diversity, GIS, citizen science

## Abstract

**Simple Summary:**

To detect changes in migrating bird populations that are usually gradual, regular counts of the flocks should be carried out. This is vital for giving more precise management decisions and taking preventive actions when necessary. Traditional counting methods are widely used. However, these methods can be expensive, time-consuming, and highly dependent on the mental and physical status of the observer and environmental factors. Taking these uncertainties into account, we aimed at taking the advantage of the advances in the artificial intelligence (AI) field for a more standardized counting action. The study has been practically initiated 10 years ago by beginning to take photos on a yearly basis in predefined regions of Turkey. After a large collection of bird photos had been gathered, we predicted the bird counts in photo locations from images by making strong use of AI. Finally, we used these counts to produce several bird distribution maps for further analysis. Our results showed the potential of learning computers in support of real-world bird monitoring applications.

**Abstract:**

A challenging problem in the field of avian ecology is deriving information on bird population movement trends. This necessitates the regular counting of birds which is usually not an easily-achievable task. A promising attempt towards solving the bird counting problem in a more consistent and fast way is to predict the number of birds in different regions from their photos. For this purpose, we exploit the ability of computers to learn from past data through deep learning which has been a leading sub-field of AI for image understanding. Our data source is a collection of on-ground photos taken during our long run of birding activity. We employ several state-of-the-art generic object-detection algorithms to learn to detect birds, each being a member of one of the 38 identified species, in natural scenes. The experiments revealed that computer-aided counting outperformed the manual counting with respect to both accuracy and time. As a real-world application of image-based bird counting, we prepared the spatial bird order distribution and species diversity maps of Turkey by utilizing the geographic information system (GIS) technology. Our results suggested that deep learning can assist humans in bird monitoring activities and increase citizen scientists’ participation in large-scale bird surveys.

## 1. Introduction

Birds are excellent ecosystem service providers that can pollinate flowers, scavenge carrion, disperse seeds, devour pests, cycle nutrients and qualify the environment in benefit of other species and humans [1,2]. In this manner, birds play critical ecological roles for the health and consistency of many ecosystems as important elements [3]. Birds are also very sensitive to changes in habitat structure and composition and are good indicators of habitat quality and biodiversity [4,5,6,7]. However, recent studies indicate that likewise the most of the global biodiversity components, birds are declining in the recent years due to human-related activities [8,9,10,11]. In general, raptors are more threatened than birds—52% of global raptors populations have declined and 18% are threatened with extinction [12].

The conservation and management of bird species require both actual spatial and temporal data [13,14]. In other words, occurrence, distribution, density, status, habitat relationships, responses to environmental change and human-related activities, population trend of birds, and species composition of particular areas need to be known using bird counting and monitoring methods. This fact brings us to developing effective wildlife management plans and conservation strategies [15,16]. The spatial data on bird richness and activity is vital for these conservation activities at species, taxonomy and ecosystem level. For this purpose, the usage of GISs in bird observation has received attention in the animal ecology literature. Nandipati and Abdi [17] investigated the diversity of birds counted with a manual investigation over an area of Portugal with the use of GIS techniques. The paper mainly discusses the capability of interpolation methods that are available in the ArcGIS software package for the modeling of bird species richness overlaid on CORINE-based land cover classes. Butler et al. [18] plotted the number of Waterfowl species at each observation position and created a density map accordingly with the use of GIS software. Grenzdörffer [19] used UAV imagery to detect and count birds over the region of the Baltic sea using image classification and GIS-based post-processing methods. The method takes advantage of good contrast of the gulf and its surroundings compared to the birds which ease the detection progress and by no means can be generalized as the author mentions. Turner [20] presented a paper about a volunteer-based bird monitoring project. The outcome of the project is a GIS-based report which shows the bird species density over in and around the Tucson, Arizona, United States area. The data collection was performed with a visual investigation for each Spring period between 15 April and 15 May. Volunteered bird counting and implementation of bird tracking using GISs is also a good example of citizen science (i.e., public participation in scientific research). There is another project called Audobon The Christmas Bird Count where any person can join in the community to provide the number of birds reported from any location and the results are visualized over an online viewer [21]. Sauer et al. [22] reviewed bird map preparation methods from point count data. Gottschalk et al. [23] modeled a large-scale ecology with bird survey data, terrestrial measurements of vegetation, and creation of a habitat map with the processing of Landsat satellite imagery.

Since the counting of birds is essential in almost all bird monitoring studies, various counting techniques have been used to count landbirds over the years by ornithologists and others, including point counts, line transects, mist-netting, playback call, etc. [24]. However, the point count is the most commonly used method for on-ground landbird counting, specifically for soaring migrant birds such as storks, pelicans, and raptors [25]. The fixed point count method is widely used in monitoring avian communities and bird migration on ground [26,27]. Accordingly, the observer detects and counts birds with a binocular or telescope, waiting at suitable points where bird migration can be easily monitored [25]. However, this method is time-consuming and often needs more than one experienced observer [28]. Especially, in the migration bottlenecks where bird migrations occur intensively in specific periods, the observer has to detect and count birds and keep records. Furthermore, the success of point counts is much obliged to the human census and affected by the observer’s ability and experience, environmental and topographic variables and birds’ detectability [27]. The observer’s previous experiences on bird detection, counting, and species identification are very crucial [29]. The observer’s performance is also influenced by physical health, age, and mental and cognitive abilities such as motivation, hearing acuity, eyesight, and fatigue level of an observer [30,31]. Environmental variables are affected by both observer’s efficiency and bird behavior [32]. Detection of birds in bad weather conditions such as intense cloud cover, rainy and foggy weather, and low light intensity might not be possible [33]. Birds might also be hidden from observers in an undetectable level due to vegetation and topographic features [34]. Another issue is that the detectability of birds can be different according to their physical and behavioral attributes [35]. As mentioned above, though conventional point count is still one of the best methods for bird detection and counting, it is expensive, logistically difficult, time–consuming and needs experienced persons.

However, taking pictures with telephoto lens digital cameras (i.e., photographic count) makes observations relatively easy and may help to overcome some uncertainties [36]. Photographs can be taken when the bird is seen and/or during mass migration events, then they can be processed manually on a computer, and species, numbers, sex and offspring/adult information are more easily identified. Even so, this process not only takes a lot of time and effort but also needs experienced observers. Furthermore, in bad weather conditions (e.g., in the presence of heavy cloudiness and fog), both the quality of observation and photographing decrease and may cause errors. Since accurate detection and counting is crucial for the monitoring and management plans, the manual process may cause disruptions in the completion of the plans in time [37].

Rapidly developing machine-learning-based computer vision and image recognition methods may provide us with better solutions to overcome these problems [38,39,40]. A machine-learning system generally requires domain expertise to transform the raw pixel values of an image into a suitable feature vector for identifying objects of interest. Unlike traditional machine learning approaches, deep learning does not use hand-crafted image features that are possibly not well-suited to the problem. Rather, deep neural networks take raw pixel values and bounding boxes of objects of interest as input and automatically learns hidden image features most relevant to the application [41]. Hence, this automatic detection and counting methods are also feasible for non-experts. Chabot and Francis [42] provide a comprehensive review of bird detection and counting in high-resolution aerial images. The methods applied in the discussed papers vary from traditional image analysis techniques including thresholding and template matching to segmentation and supervised/unsupervised classification, but still, no deep learning method is mentioned. Even though papers related to machine learning applications on unmanned aerial vehicle- (UAV) or satellite-derived images have been published so far [43,44,45,46,47], the studies focusing on on-ground photography are limited [48,49]. At the same time, digital photography has entered a new era with the availability of cloud-storage and location-aware cameras and smart-phones that enable the sharing of photographs on a truly massive scale. Our work is based on the idea of exploring large collections of geo-tagged bird scene photographs purposing to monitor their population. Briefly, we focus on image-based bird population counting and mapping using on-ground digital photographs that we have collected. As such, the aim of this paper is to assess higher volumes of geospatial bird data with less errors and automate the related map generation. This aim is met by our main contributions that are threefold. First, we publish a new collection of wild bird photographs that have been taken in various environments on a regular basis and may lead to the construction of machine-learning models for birds. Second, we employ object detection models utilizing deep neural networks to help automate the process of bird counting that may encourage the avian science community for more efficient ways of evaluating the local bird ecosystem. Lastly, we use the model outputs to provide a spatial mapping of birds over the last decade as a step towards more rapid and reliable bird population mapping.

## 2. Materials and Methods

**Figure 1:**
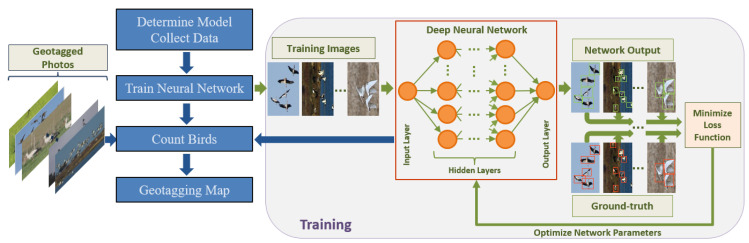
(**Left**) General workflow of the study including model determination and data collection, model training, bird counting, and map preparation. (**Right**) Typical flowchart for the deep neural network training process in which the network adjusts its parameters to match the input image bounding box predictions to the ground-truth boxes using a given loss function. The details of all steps are presented in the following sections.

Deep learning models need training data to learn how to use image pixel values to convert to the target output [41]. Hence, the input to the training procedure is a set of bird-scene images and the bounding boxes enclosing the birds inside. The output is a deep neural network trained by optimizing various parameters that minimize the loss function between the network’s output and the corresponding training bounding boxes. After the training phase is complete, the neural network is fixed and ready to blindly predict the birds’ locations and sizes in new input images that are not necessarily from the same scenes that the network has been trained on. Finally, we use this predictive power to map local birds’ diversity distribution. An overview of our approach is shown in Figure 1. The rest of this section is organized in the same processing order.

### 2.1. Data Collection

We have photographed 3436 natural bird scenes for the purpose of researching a comprehensive bird data collection that contains a large number of species with high within-species and low between-species variance. All photos also hold GPS coordinates as metadata. The full photo collection [50] can be downloaded from the Kaggle data repository https://www.kaggle.com/dsv/1193435. There can be from several to thousands of birds per photo summing up to more than 110,000 birds in all photos. The photos were taken at 21 different observation points (OPs) in 12 different cities in Turkey where the majority are migration bottlenecks (i.e., Tekirdağ–Kıyıköy (OP1), Vize (OP2), Balabanlı (OP3), Yalova–Çakıl (OP4), Armutlu (OP11), Afyonkarahisar–Dinar(OP6), Ankara–Sırçasaray barrage OP(21), İstanbul–İstanbul garbage dump (OP7), Çataltepe (OP16), Ömerli wind energy power plant (OP19), Balıkesir–Marmara Island (OP8), Keltepe (OP18), Poyraz Lake (OP20), Çanakkale–Gelibolu-Saros (OP9), Bursa–Harmanlık (OP10), Hatay–Belen (OP13), Şenköy (OP14), Atik (OP15), Mersin–Mut (OP5), Sivas–Kangal-Mağara (OP12), Kırşehir–Geycek (OP17) and at arbitrary times in Spring and Autumn seasons during 10 years period from 2010 to 2019 using ground-based methods. The properties of the camera mostly used in this study are given in Table 1.

**Figure 2:**
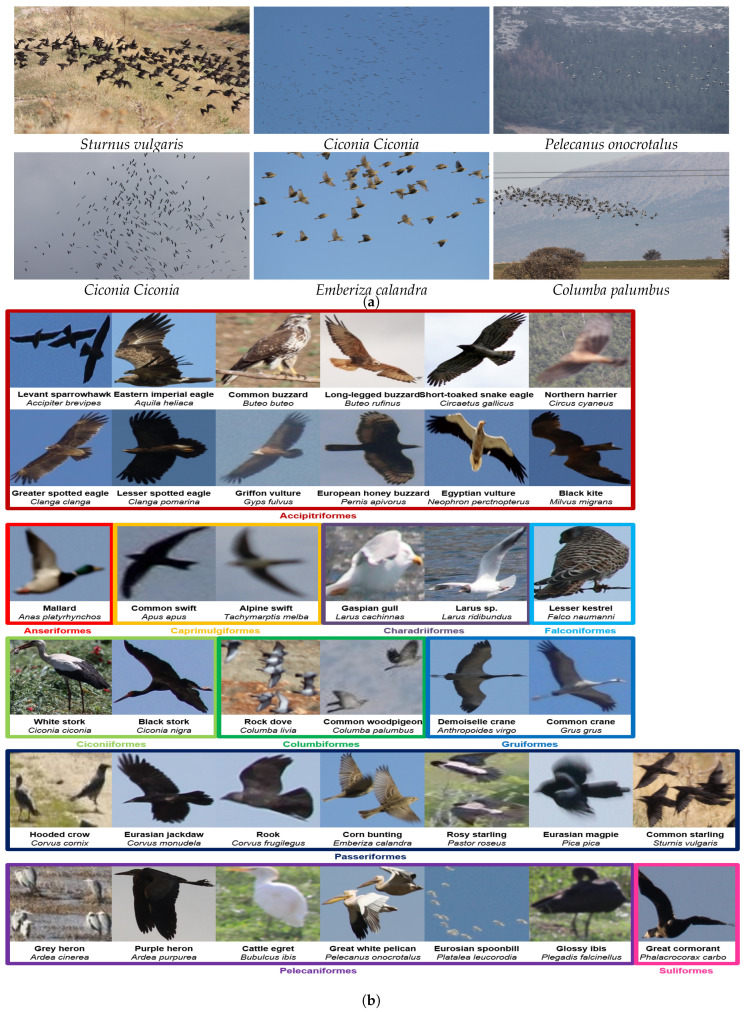
(**a**) Example 3456 × 5184 geo-tagged photos. Species of the birds inside each image are given. (**b**) Example images of 38 observed species grouped by their order names. Common and scientific names of each species are given in bold and italics, respectively. Close shots of several species could not be taken owing to their flying habits. Best viewed in zoom and color.

Photos have been captured at different resolutions and from different viewpoints. In case it was impossible to photograph all birds in an area with a single shot, multiple photos were taken to span the entire flock of birds while keeping as little overlap as possible across those photos. There exist different backgrounds depending on observation environments, such as the vicinity of water surfaces, beaches, forests and farmlands, hosting various bird habitats, bird activities (e.g., flying birds lead to flat sky backgrounds) and weather conditions. Bird poses may also differ according to lateral or frontal directions and actions such as flying, standing straight and bending. Example images from our bird scene photo collection are shown in Figure 2a. Moreover, each bird might be belonging to one of the following 38 identified species under 11 different orders as shown in Figure 2b.

### 2.2. Automated Bird Detection

Having photos containing bird populations as well as their location and time-stamp information at hand, our goal is to examine different machine learning methodologies for detecting birds in these photos. Here, we provide an overview of how deep neural networks can be used to detect every bird inside an image. Examples of similar techniques have been for detection of marine birds from videos [51], tracking of Serengeti wild animals in camera-trap images [52], detection of wild birds using UAV imagery [53], detection of Interior Least Tern in uncontrolled outdoor videos [54]. A deep neural network represents a relatively complex hypothesis function that maps features (e.g., image pixels) to the desired output (e.g., pixel class labels for classification, bounding boxes for detection) by computations in multiple layers. A network takes an input image as its input layer (i.e., each pixel is a neuron) and passes information from one neural network layer to the next until the last layer which corresponds to the hypothesis function output [41].

**Figure 3:**
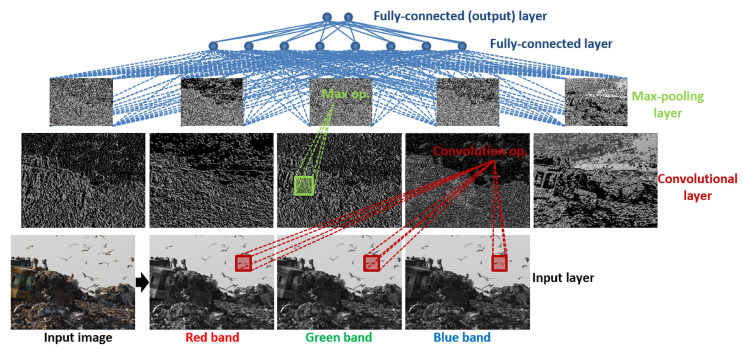
A basic neural network with three hidden layers for image analysis—a.k.a. convolutional neural network (CNN). Input layer neurons correspond to the pixels in red, green and blue bands of the original image taken in İstanbul garbage dump. Each pixel in the convolution layer corresponds to a neuron output which is computed by one of the learned convolution filters applied to the neighborhood of that pixel in the previous layer. Rectangular images in max-pooling layer contain pixels (a.k.a. neurons) each of which is the maximum of its neighborhood in the previous layer. Information flows bottom-up from lower-level features to more abstract ones. Fully-connected layer neurons collect information from all neurons from the max-pooling layer and output layer neurons are expected to give the desired output values.

Deep neural networks can be made of several different types of layers, each of which is made up of a fixed number of artificial neurons that jointly act as input to the next layer neurons [55]. Typically, types of hidden layers (i.e., layers except the input and output layers) used for image analysis include (Figure 3)

- convolutional layers each of which convolves the previous layer’s neuron outputs by different convolution filters (i.e., each filter is a linear combination of neighboring neurons inside a fixed-size window) and then apply a non-linear function,
- max-pooling layers each of which outputs the maximum of input neuron values in each grid when the input neurons are partitioned into non-overlapping rectangular grids,
- fully connected (FC) layers where each neuron’s output is computed as a non-linear function applied to a linear combination of outputs of all neurons in the previous layer.

Each convolutional layer produces feature maps as separate feature images based on the previous layer [56]. The last feature map is generally assumed to have relevant and meaningful information regarding the input object-of-interest. Pooling layers down-sample the previous layer keeping the most important pixels. Finally, fully connected layers produce the final hypothesis output. Image pixels form the input layer (i.e., convolutional layer) neurons and the output layer (i.e., a fully-connected layer) neurons are the desired values such as the location and the size of a bird. Given a set of training images with the true bounding boxes, learning the weight and bias parameters that are used in computing linear combinations at each layer are posed as a regression problem that is solved through a stochastic gradient-descent (SGD) based optimization algorithm called error back-propagation [55]. The training flow is illustrated in Figure 1. The goal is to find a set of network parameters that give a local minimum of an arbitrary loss function such as cross-entropy loss for binary outputs and mean-square error for continuous outputs. The loss function compares the output of the current network with the expected outcome. After initialization, the network parameters are updated in the reverse direction of the gradient of the loss function until the loss converges.

In our study, the so-called Faster R-CNN architecture [57]—a variant of CNN designed for generic object detection—was used to map image pixels to the corresponding output bounding boxes containing birds. Readers are referred to the original paper for more technical details.

#### 2.2.1. Faster R-CNN Network Architecture

**Figure 4:**
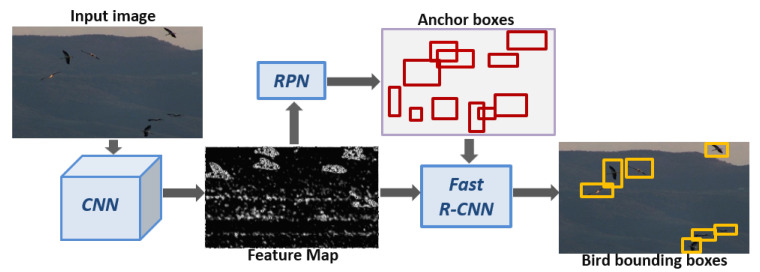
Faster R-CNN architecture. The input to the algorithm is a bird scene image. Firstly, a CNN generates a context-aware feature image on which the Regional Proposal Network (RPN) generates candidate regions of interest (ROIs). Inside each candidate, a Fast R-CNN is run to decide whether that ROI contains a bird and if so, adjust its coordinates and scale. The species of the birds inside the image is *Ciconia ciconia*.

Faster R-CNN consists of a feature extraction network (i.e., convolutional layers) followed by two sub-networks that are called Regional Proposal Network (RPN) and Fast R-CNN detector. The feature extraction network extracts a feature representation of the image through a series of image convolutions. The RPN is used to generate regions of interest (ROIs) from this feature map where objects are likely to exist. These ROIs serve as candidate object proposals that are classified into their actual classes by the Fast R-CNN detector. The architecture is illustrated in Figure 4.

##### Feature Extraction Network

**Figure 5:**
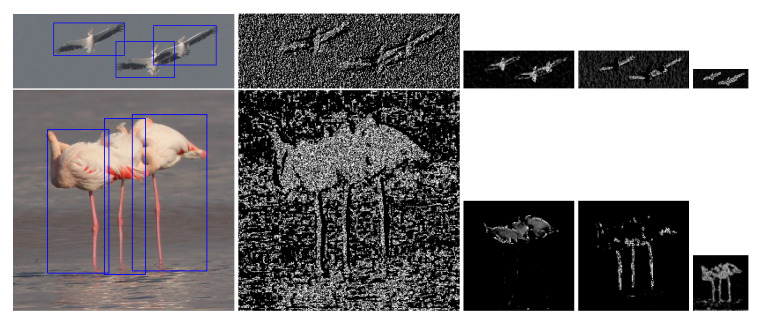
Visualization of the network layers. Each row represents a separate example image. Bird species are Pelecanus onocrotalus (**up**) and Phoenicopterus roseus (**bottom**). From left to right: input image with detected birds, example feature map images from 1st, 2nd, 2nd and 3rd convolution layers. Feature maps are scaled according to their original sizes.

The feature extraction network is a fully convolutional neural network (CNN) to extract a feature map of an input image through a series of image convolutions that have been trained to enhance different bird features [56]. The learned convolutional filters provide hierarchical levels of abstraction of the input birds. Figure 5 illustrates example layers of the feature extraction network whose filters are trained with our data set. Lower network layers caught the general features such as edges and blobs and then, upper layers captured more specific features such as wings, legs, median body and beak and finally, more semantic features such as the whole body.

##### Region Proposal Networks

A Region Proposal Network (RPN) takes as input the last convolutional feature map from the feature extraction network and generates as outputs a set of rectangular object proposals, each with an objectness score, through a series of FC and max-pooling layers [57]. A spatial window was slid over the feature map and at each sliding position of the feature map, *k* boxes (anchors) at different scales and aspect ratios are used as candidate object proposals. These anchors may or may not contain objects of interest. For each anchor, the RPN predicts a likelihood of containing an object of interest as well as two offsets and a scale factor which refines the object’s position. The refined anchors are sorted by their likelihoods, subjected to a non-maximum suppression and the ones having the highest likelihoods are fed into the Fast R-CNN detector as object proposals.

##### Fast-RCNN Detector

After RPN, the Fast R-CNN detector takes anchors with different sizes and extracts a fixed-length feature vector for each anchor through an RoI pooling layer [58]. This enables the use of CNN feature maps of the same size for all anchors. Each feature vector is then fed into consecutive FC layers. The final outputs of the detector are the final binary class label for indicating the existence of a bird and final regressed bounding box coordinates.

#### 2.2.2. Data Augmentation

Despite its success in image recognition, deep learning models need lots of images to be trained in many problems, especially when the network size and data variability are large [59]. Limited training data may cause the over-fitting problem [55] in a neural network especially when the network has to generalize across a diverse set of samples such as birds. In that case, significantly more data is required to learn a reliable set of network parameters. To overcome this problem, the feature extraction network can be pre-trained using another available big data set, with possibly different object categories, so that the training can continue from those learned parameters rather than random values using our smaller data set of bird images. This technique for compensating the negative effect of limited training data is called transfer learning [60]. Other solutions that we applied to help improve the performance were to augment the image data set by flipping the images horizontally [55] and drop-out regularization which refers to deactivating randomly chosen neurons during training [61].

#### 2.2.3. Performance Evaluation

The first set of experiments was performed to evaluate the accuracy of the automatic bird detection algorithm. In this section, we describe the experimental settings and present quantitative and qualitative results.

##### Data Set

First, a subset of the collected bird scene images in Section 2.1 was chosen. Then, we tagged every bird inside every image in this subset by an enclosing bounding box to create a new bird detection data set. For the experiments, the resulting data set was divided into two subsets: training set (i.e., to train a detection model) and test set (i.e., to evaluate the trained model). The resulting training and test sets consisted of 3283 birds in 491 images and 1602 birds in 156 images, respectively. It can be seen in Figure 2b that there exist large intra- and inter-species variations in terms of appearance, pose, scale, and the number of birds across images making this data set challenging. The data set can be accessed through the following private link: https://www.kaggle.com/dsv/1193435.

##### Experimental Protocol

In order to fit images in GPU memory, we divided them into overlapping tiles such that their height is 600 pixels and their width is the original image width. If a cropped sub-image had a height shorter than 600 pixels, zero-padding was applied. The number of RPN proposals per image was set to 300. To deal with varying ranges of bird sizes and shapes, we considered k=18 anchor boxes for each pixel using six scales with box side lengths of 16, 32, 64, 128, 256 and 512 pixels and three aspect ratios of 1:1, 1:2, and 2:1. A deep learning image recognition application usually requires thousands of images to train the model, as well as a graphics processing unit (GPU) to rapidly process this huge amount of data. The experiments were conducted on a system with an NVIDIA GeForce GTX-1050 GPU, Intel Core i7-7700, 3.60 GHz CPU and 8 GB RAM.

##### Baselines for Comparison

We compare the Faster R-CNN approach against two baseline methods: bag-of-words (BOW) [62] and single-shot-detector (SSD) [63]. The former is a traditional object recognition method that utilizes a support vector machine classifier with Scale Invariant Feature Transform (SIFT) features [64]. On each image patch obtained from running sliding windows of predetermined sizes and aspect ratios on the input image, a histogram of K-means quantized SIFT features is constructed which is then fed to a support vector machine (SVM) for classifying the patch into bird or background classes. The latter is a more recent approach that uses the same convolutional layers as Faster R-CNN but then, does not undergo the RPN step and each anchor directly predicts the existence of a bird. However, SSD predictions are computed across multiple feature maps (i.e., convolutional layers) instead of using only the last convolutional layer. For both Faster R-CNN and SSD architectures, we utilized Tensorflow Object Detection API [65] in Python available at https://github.com/tensorflow/models/tree/da903e07aea0887d59ebf612557243351ddfb4e6/research/object_detection. The scripts to replicate the Faster R-CNN experiments using the published data set can be found at https://github.com/melihoz/AUBIRDSTEST. The already existing feature extraction network Inception-v2 [66] which has 13 convolutional layers and pre-trained with the Oxford-IIIT pet data set [67] was utilized for transfer learning. For the BoW model, the C++ implementation available at https://github.com/royshil/FoodcamClassifier was utilized.

##### Evaluation Criteria

Quantitative evaluation was performed in the test set by comparing the binary detection maps, obtained by applying a uniformly sampled range of thresholds on detection scores, to the validation bounding boxes. Each detection for a particular threshold is considered to be positive if its intersection-over-union (IoU) ratio with a ground-truth annotation is greater than 0.5. By setting each threshold for detection scores, a set of true positives and false positives can be generated to calculate precision and recall that have been commonly used in the literature to measure how well the detected objects correspond to the ground truth objects. Precision is computed as the ratio of the number of correctly detected birds to the number of all detected birds while recall is computed as the ratio of the number of correctly detected birds to the number of all birds in the validation data. We also found the F-measure which combines precision and recall as their harmonic mean.

### 2.3. Bird Counting

In this section, we present a bird counting test in order to see the effectiveness and usability of automated counting over the traditional manual method. We used 45 of our collection of photographs for the experiments. The photographs were selected so that the sample would be as much representative as possible in terms of different bird species, flock size, and environmental conditions of the photo (e.g., presence of fog and obstacles such as trees). We compared the bird counting performances of the following three methods:

- Manual: Two experts, Exp1 and Exp2, that have PhDs in ornithology and at least five-year field experiences on bird counting, separately counted the photographs in random order on a computer monitor. In this method, experts usually count birds by grouping them (i.e., not one by one, but five by five or ten by ten). The maximum allowed duration of effort for each photo was fixed at 3 min.
- Automated: This method corresponds to the Faster R-CNN model output.
- Computer-assisted: We also evaluated the performances of the two experts’ manual countings with the aid of the model outputs. Each expert was shown on the screen the automated detection result image of each photo and asked to improve the overall count by adding and subtracting uncounted and over-counted birds, respectively. Counting processes were held in the same conditions with the manual method two weeks after the manual count.

Three different evaluation metrics were recorded for each method and photo: bird count, duration of counting effort and count error. The count error for each photo was calculated as the ratio of the absolute difference of the correct number of birds and the resulting count to the correct number. For instance, if an expert and/or the model counted 80 birds out of 100 correct number of birds, then the error rate would be 20% or 0.20. On the other hand, if 125 birds were counted, the success rate would be 0.25. Q-Q plots were used to check whether the observations of each metric were normally distributed or not. Since the bird count metric was not normally distributed, correlations between the correct number of birds and manual, automated and computer-assisted counts were examined with non-parametric Spearman’s rank test. We also used the Kruskal–Wallis rank sum test for testing whether bird count, duration and count error values of the three considered methods originated from the same distribution [68]. For post-hoc comparisons, the Dunn test was used. Epsilon square $\epsilon^{2}$ was computed as an estimate of effect size [69]. We used the Wilcoxon signed-rank test on paired samples to test the difference between manual and computer-assisted counting efforts with respect to each metric. The Wilcoxon effect size (*r*) was calculated as an estimate of effect size. All statistical tests were performed using R v. 4.0 [70] and the additional packages rstatix v0.5.0 [71], FSA v0.8.30 [72], rcompanion v2.3.25 [73], the tidyverse v1.3.0 [74].

### 2.4. Geospatial Bird Mapping

As a case-study, we prepared two different spatial bird maps: one shows the distribution of the observed bird orders and the other to shows the diversity of the observed bird species at the OPs. For this aim, we used the corrected model outputs to gather spatial data on bird diversity and bird abundance on each OP. To summarize, the model output photos were checked to correct the bird counts and birds’ species and orders were determined by an expert in ornithology. Photos were assigned to the OPs according to their geo-locations. For the order distribution map, we grouped the bird species into 11 different orders and approximated the total number of birds belonging to each order at all seasons between the years 2010 and 2019 by summing up the semi-automated photo counts. The OP coordinates were set as the point features and the total count for each order at each point was assigned as one of the attributes for that OP. The bird order categories are shown in Figure 2b. As well, a presence-only species diversity map was established to see how many different species showed up at each OP during the last 10 years.

## 3. Results

### 3.1. Automated Bird Detection Results

**Figure 6:**
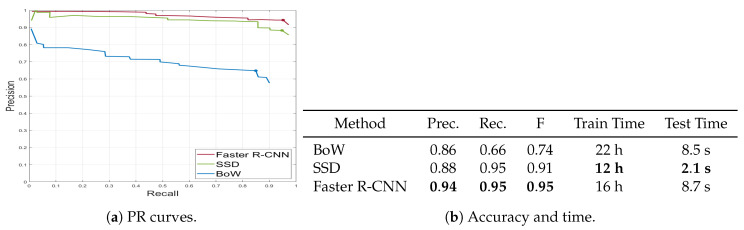
Summary of detection results for BoW [62], SSD [63], and Faster R-CNN [57] (best viewed in color).

**Figure 7:**
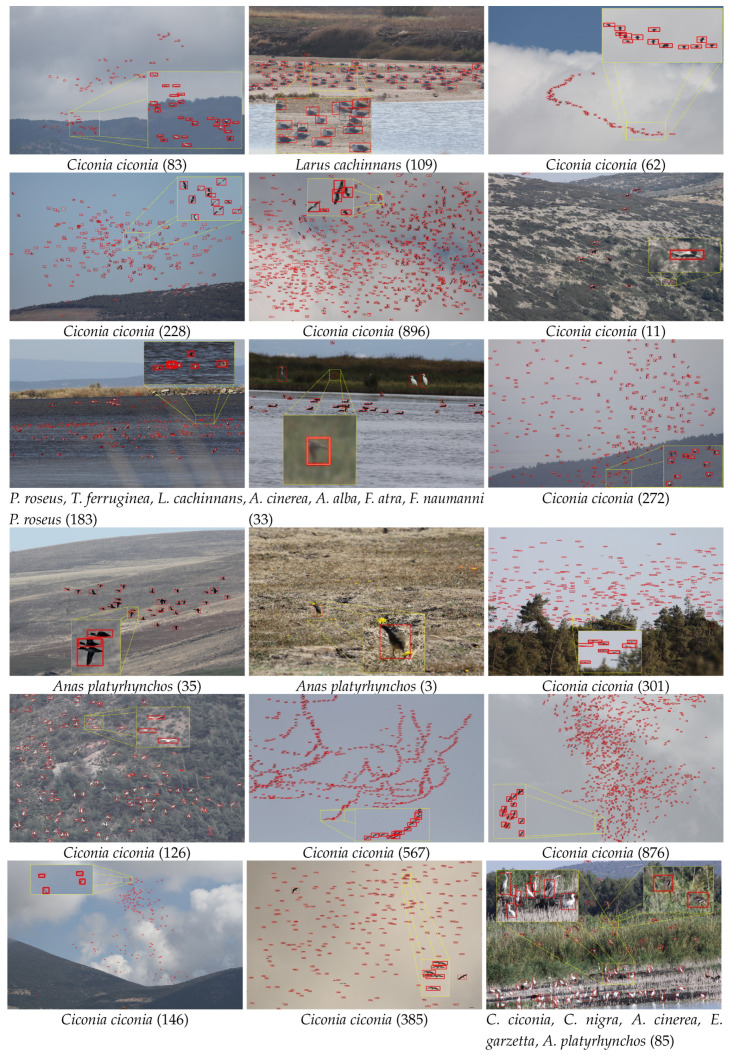
Example bird detection results with the species and the number (in parentheses) of detected birds (best viewed in zoom and color). Zoomed areas illustrate the local details for different bird appearances and arrangements.

**Figure 8:**
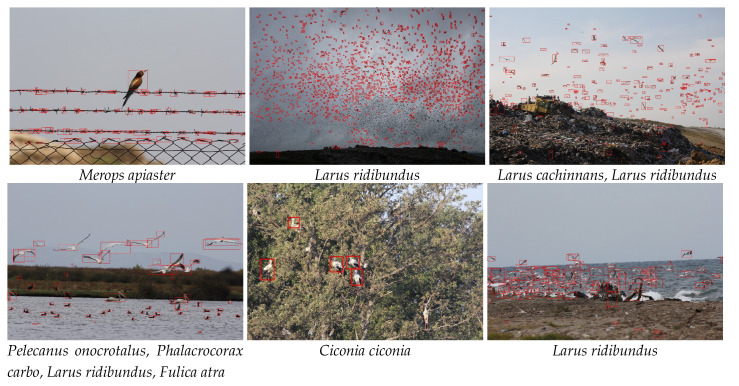
Example erroneous detection scenarios with the species of detected birds (best viewed in zoom and color). **Upper left:** False detections due to shape similarity. **Upper middle:** Misdetections due to small bird sizes. **Upper right:** Misdetections due to occlusion by trees. **Lower left:** Mis- and false detections due to background clutter. **Lower middle:** Mis- and false detections due to complex background texture. **Lower right:** Mis-, false and over-detections due to high level of overlap.

Figure 6 shows the precision-recall curves for the above-mentioned algorithms as well as a table summarizing the accuracies giving the best F-scores and computational times. We further demonstrate qualitative detection results in Figure 7. Additionally, we analyzed different sources of errors in Faster R-CNN detections (Figure 8) that also caused count errors.

### 3.2. Bird Counting Results

**Figure 9:**
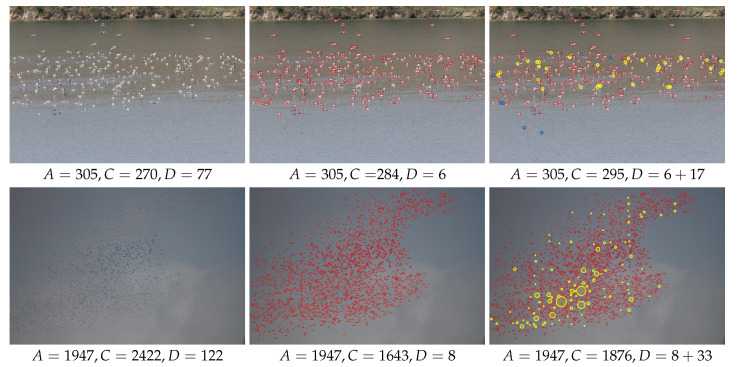
Counting examples (best viewed in zoom and color). Each row represents a different image. Bird species are *Larus cachinnans* (**up**) and *Ciconia ciconia* (**bottom**). **Left:** Original image and manual counting stats in sub-caption. *A*, *C* and *D* denote the correct number of birds, the resulting count, and the duration in seconds, respectively. **Middle:** Automated detection and counting stats in sub-caption. **Right:** Computer-assisted counting and stats in sub-caption. Yellow circles correspond to manual additions to the automated count (radius of each circle is proportional to the number of group count by the expert in that area) and blue circles correspond to manual subtractions from the automated count.

Figure 9 demonstrates examples for manual, automated and computer-assisted counting methods. Experts mostly grouped birds, as if they were doing a point count in photos, while counting with the manual and computer-assisted methods. However, proceeding from the automated detection results gave them more time to catch the individuals that might have been missed by the point count.

**Figure 10:**
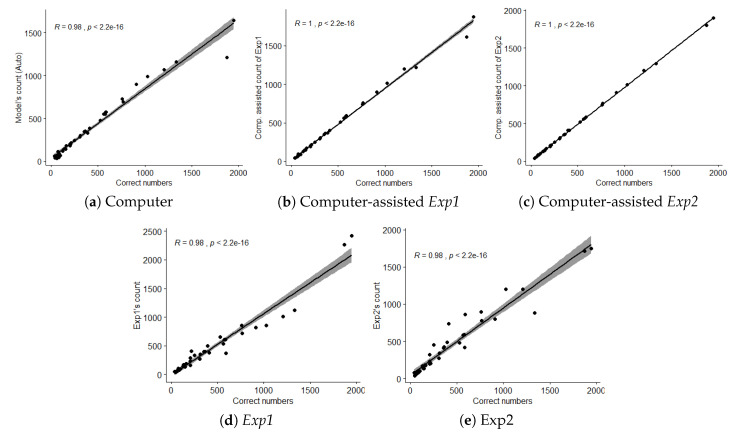
Count correlation graphs (Best viewed in zoom).

Both experts’ and model’s counts were positively correlated with the actual bird counts (Figure 10). We found no difference in bird count ($p=0.98,x^{2}=0.36,\epsilon^{2}=0.01$) but significant differences in count duration ($p=0.001,x^{2}=170.94,\epsilon^{2}=0.76$) and count error ($p=0.001\%,x^{2}=81.56,\epsilon^{2}=0.36$). Additionally, paired comparison of the results of manual and computer-assisted methods revealed no significant difference in bird count metric (p=0.11,V=341,r=0.24). On the other hand, significant differences in count duration (p=0.001,V=0.1,r=0.87) and count error (p=0.001,V=40,r=0.76) were observed.

**Figure 11:**
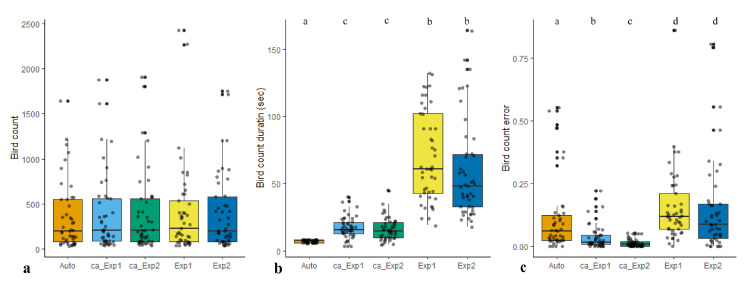
Box plots corresponding to (**a**) bird count, (**b**) duration and (**c**) count error by the computer, computer-assisted Exp1 and computer-assisted Exp2, Exp1 and Exp1, respectively.

Corresponding box plots for all metrics are shown in Figure 11. All of the descriptive statistics for test variables are given in Table 2.

**Figure 12:**
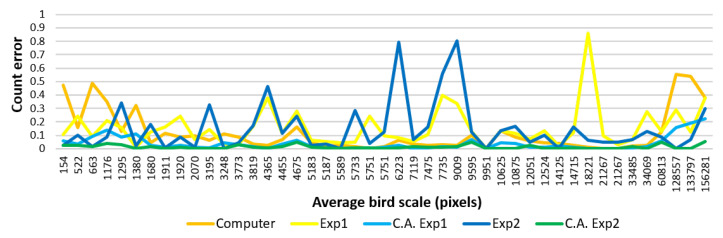
Average bird size versus count error graphs (best viewed in color). Computer-assisted is abbreviated with C.A.

We also investigated the effect of the scale of the birds in the image on counting performances (Figure 12). The scale of a bird corresponds to the number of pixels it occupies and depends on the camera settings, size of the bird and camera shot distance.

### 3.3. Bird Mapping Results

**Figure 13:**
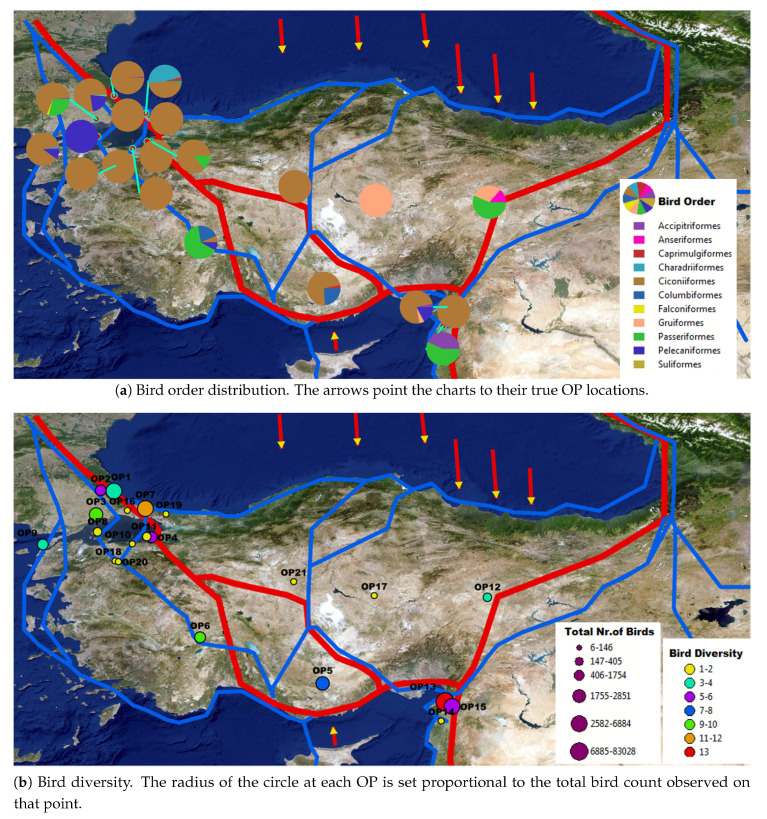
Bird order distribution and diversity level map of Turkey with overlays of geographic information system (GIS) bird migration routes data layer extracted by Turan et al. [79] (best viewed in zoom and color). The red lines represent the primary migration routes where the magnitude of the bird migration is higher, the blue lines represent the secondary migration routes where the magnitude of the bird migration is relatively high and lower than the primary routes, and arrows represent the broad front across routes of migrant birds where the bird movement occurs on north–south axis rather than following the particular routes. ArcGIS software was used for visualization.

Figure 13 shows the resulting geo-visualizations of the bird order distribution and species diversity observed in the last decade as pie-charts over the physical map of Turkey (Figure 13). Observed bird counts for each species at each OP used to prepare these maps are given as a supplementary table (Table S1). Although our results include only photo-based bird numbers (i.e., not the entire seasons’ bird monitoring data), bird map is still informative for the bird diversity and bird movement activity on OPs. Eighteen species were soaring migrant birds. The most numerous and most common species was *Ciconia ciconia* which migrates in large flocks even over entire Turkey. Hatay–Belen had the highest bird species diversity and bird count among the other OPs, which is known as an important migration bottleneck for bird migrations in the Western Palearctic [75,76]. The primary routes of the north–south migratory bird movements through Turkey occur between the Eastern Black Sea (Borçka) and Thrace region (Bosporus) in the north and the Mediterranean region in the south (Belen) [77,78]. As expected, bird diversity and count were higher in those OPs that were near the primary routes.

## 4. Discussion

### 4.1. Automated Bird Detection Discussion

The quantitative results (Figure 6) showed that the deep neural networks that use data-driven features outperformed the BoW that manually extracts features from sliding windows. Lower recall despite comparable precision for BoW can be explained by false detection of many non-bird regions as birds. Although SIFT was the most popular feature extraction tool during the previous decade, we observed that the hand-crafted SIFT features could be noisy for natural scenes and could not encode shape information distinctive to birds. Hence, many patches that did not correspond to birds appeared in the output. The slightly lower accuracy performance of the SSD compared to the Faster R-CNN can be explained by the inherent extreme bird/background class imbalance problem encountered in one-shot detection schemes (i.e., without an RPN) during training [80]. That is, the last prediction layer in SSD was exposed to many candidate ROIs, only a few of them containing birds. This spent most of the training effort mostly on adjusting the network parameters to avoid these easy negatives rather than fine-tuning them in favor of the bird examples. Conversely, the RPN in Faster R-CNN filtered many background instances out resulting in a more balanced bird/background ratio.

The visual results in Figure 7 showed that the Faster R-CNN was successful in identifying the regions corresponding to birds and could cope with difficult situations with multiple overlapping birds and birds with extreme poses. Furthermore, the computer-based model could detect camouflaged birds that were almost indistinguishable from their backgrounds.

Major misdetections occurred due to very small bird sizes (Figure 8). As the distance to birds increases, such birds often correspond to very small details (i.e., several pixels) for even the human eye to differentiate. Consequently, these birds might have been lost in the low-resolution feature map that was generated by the last convolutional layer after several max-pooling operations. Small object detection is also an open-problem in many other datasets [81]. Other main reasons for the misdetections were intra-species variation, unusual bird poses, occlusions by other birds and plants, cast shadows, and background clutter. The reason for most false detections were non-bird regions that had similar color and shape characteristics to birds. The last type of error, called over-detection, is defined as a single bird being divided into multiple bounding boxes and was mostly seen in larger birds. Nevertheless, unless a bird is not totally missed or divided into multiple parts, the localization ability of the algorithm is not as critical when the goal is counting. In the extreme case, a single correctly detected pixel inside a bird is sufficient for counting and can attract one’s focus-of-attention to that bird.

The last column in the table in Figure 6b shows the average running times over all images for the three algorithms. The quantization step (i.e., clustering the high-dimensional SIFT feature vectors) took a very long time in BoW training. SSD achieved the fastest test performance whilst Faster R-CNN the worst since the RPN and the prediction stages are fully consecutive in SSD reducing redundant computations. The results revealed that in case of real-time bird detection (e.g., from surveillance videos), Faster R-CNN would fail with its three-step architecture.

We believe that the output of the automated methods can be useful when the goal is faster and more accurate bird counting particularly when we do have lots of images but a few human volunteers. To sum up, Faster R-CNN should be preferred over the other methods if the main concern is localization accuracy. However, If the priority is speed, SSD seems a better choice. Nonetheless, the detection accuracy can be improved possibly by increasing the number of tagged images—a task which can even be organized as a citizen volunteered event, as will be discussed in Section 4.3.

### 4.2. Bird Counting Discussion

Both experts and the automated method approximately counted a similar number of birds from photos whereas the model was significantly faster than the experts. This implied that the computer-based model worked as effectively as the traditional on-ground point count method. In computer-assisted counting, the experts’ task was to correct the overall count in each photo by finding out the uncounted and/or over-counted birds in the corresponding model output. On average, tuning the model counts by an expert reduced the manual and automated count errors by factors of 5 and 4, respectively, while the total computer-assisted counting duration (i.e., automated plus correction) was still three times faster than the manual method. Thereby, computer-assisted counting appeared to be a more accurate method than manual or automated methods. Automated methods can be considered as the most technically acceptable methods since they estimate not only more accurate counts but also with the same level of precision every time the counting is repeated.

The individual graph of the automated method in Figure 12 highlighted a direct relation between the scale of a bird and the possibility of counting it. That is, the computer model gave higher error rates for very small (<1500 pixels) and very big (>60,000 pixels) scales. This was expected because these tails corresponded to rare observations that were not usually tuned by the model parameters. In particular, smaller birds were usually so invisible in the last convolutional layer of Faster R-CNN as to cause under-counting whereas larger birds were usually divided into parts that caused over-counting. SSD attempted to solve this scaling problem by computing the predictions across several feature maps’ resolutions [63], but still, the tendency of missing small objects remained and is left for future work. According to the camera settings in Table 1, a bird 750 m away from the camera with a 1 m wingspan would have covered an area of approximately 1500 pixels in the photo. This means that shooting a flock of 1 m wing-spanned birds more than 750 m away might even cause the model to miss the whole flock. The manual counting performances showed no explicit correlation with the bird scale. This suggested that factors like heterogeneity of the background and physical and/or mental discomfort of the experts may have affected the human performance at all scales. However, manual correction seemed to well compromise the computer model for extremum scales while, in between, the computer model decreased the uncertainty in manual counts.

### 4.3. Bird Mapping Discussion

When doing statistical analysis about birds, the data source should be as free of bias as possible to make more confident inferences about the whole population. A larger data size by increased count duration and number of OPs generally increases the likelihood that the data accurately reflects the target population [82]. In our case, the species distribution map in Figure 13 was prepared with 111,511 counted birds. In order to reach this number, nearly 20,000 birds had to be photographed every year on the average. In this sense, the species diversity, population size, and spatio-temporal nature of flocks’ movements make representative bird monitoring a hard challenge. In practice, this issue can be converted into a problem of citizen-scientists that can have greater time and area coverage as well as more linkage with the surrounding ecosystem especially to find out the bird migration patterns. On a single day, tens of thousands of people from all around the world go bird-watching as a scientific sport and record their photos. This big amount of data can be utilized for bird habitat assessment by the collaboration of citizen scientists, ornithologists, computer engineers, and geomatics engineers. Even so, one important bottleneck in integrating all of these data to geospatial technologies is the difficulty of counting birds. Our results demonstrated that the automated model outputs could be useful for the spatial data gathering for the bird migration over Turkey and replace the traditional point pattern analysis in GIS applications. In such a monitoring activity scenario, anyone can upload their on-ground photos at any time where the number of individuals can be extracted in a semi-automated manner. Species identification can be done by a registered society of ornithologists or bird categorization algorithms [83] that need a large data set of bird image patches with category annotations that can also be supplied by citizen scientists. However, fine-grained object classification still remains under computer-scientific level of interest because of very small details that differentiate between species and specifies an interesting direction of our future work. In this manner, a digital network of local volunteers, usually at the country level, can easily prepare their own bird distribution presence-only maps similar to our case-study demonstrated in Figure 13. This volunteered effort is prone to biases and limitations related to variability in species, time, locality, and volunteer skills [84,85]. However, spatial bias can be degraded to a certain degree using existing geospatial analytical methods [86] and consensus of the multiple automated point counts can be a natural validation method for the individual counts. Availability of photo-based counts increases the number of volunteer investigators (i.e., observation units) which increases statistical representation power. Thus, maps prepared with personal bird photo scene collections and/or Internet photo sharing sites can be compared among themselves and be used as indicators of the bird population trend in an area over time. The resulting system can also enable the users to browse and see the source photos together with the detected birds with which the maps were built.

## 5. Conclusions

We studied the automation of the bird counting problem in geo-tagged digital photos, that were taken over a long period of time in a wide area of migration routes over Turkey, using a deep learning approach. Our experiments revealed that neural networks can be more helpful in assisting humans rather than fully automating the decision as most AI systems do. These algorithms can facilitate numerous bird monitoring activities such as on-ground bird counting at migration bottlenecks, aerial bird counting, and ornithological monitoring at power plants. The model outputs were also exploited as a proof-of-concept GIS application to map the country-wide bird distribution which can inspire deeper multi-spatial and multi-temporal bird population trend analysis. In a broader sense, this multidisciplinary work can be a simple yet important step to a large-scale bird mapping activity using volunteers and citizen scientists.

## Figures and Tables

**Table 1:** Typical camera settings used in photographing birds.

**Imaging camera**	Canon 7D Mark II body + Canon EF 100–400 mm f/4.5–5.6 L IS II USM telephoto zoom lens
**Resolution**	5184×3456 pixels
**Focal length**	200 mm
**Sensor size**	22.4 mm × 15 mm
**Shutter Speed**	$\frac{1}{1000}$ s

**Table 2:** Descriptive statistics of test variables. Computer-assisted is abbreviated with C.A.

Variable	Counter	Min	Max	Median	q1	q3	iqr	Mean	sd	se	CI
*Count*	Exp1	39	2422	231	83	533	450	412.02	510.07	76.04	153.24
	Exp2	38	1748	203	85	581	496	404	430.77	64.22	129.42
	Automated	34	1643	201	79	551	472	355.33	380.08	56.66	114.19
	C.A. Exp1	42	1876	204	88	559	471	380.93	431.71	64.36	129.7
	C.A. Exp2	42	1902	205	82	559	477	390.62	450.26	67.12	135.27
*Duration*	Exp1	19	132	61	43	102	59	70.13	33.49	4.99	10.06
	Exp2	18	164	48	33	72	39	59.33	36.55	5.45	10.98
	Automated	6	8	8	6	8	2	7.29	0.97	0.14	0.29
	C.A. Exp1	4	40	16	13	21	8	17.42	8.08	1.2	2.43
	C.A. Exp2	5	45	15	10	21	11	16.4	8.46	1.26	2.54
*Error*	Exp1	0	0.86	0.12	0.07	0.21	0.14	0.16	0.15	0.02	0.05
	Exp2	0	0.81	0.09	0.03	0.17	0.14	0.15	0.19	0.03	0.06
	Automated	0	0.55	0.06	0.02	0.12	0.10	0.12	0.15	0.02	0.05
	C.A. Exp1	0	0.22	0.01	0.01	0.04	0.04	0.04	0.05	0.01	0.02
	C.A. Exp2	0	0.05	0.01	0	0.02	0.02	0.01	0.02	0	0.01

## Supplementary Materials

The following are available online at https://www.mdpi.com/2076-2615/10/7/1207/s1, Table S1: Observed bird counts for each species at each OP.
